# Acute mesenteric ischemia: updated guidelines of the World Society of Emergency Surgery

**DOI:** 10.1186/s13017-022-00443-x

**Published:** 2022-10-19

**Authors:** Miklosh Bala, Fausto Catena, Jeffry Kashuk, Belinda De Simone, Carlos Augusto Gomes, Dieter Weber, Massimo Sartelli, Federico Coccolini, Yoram Kluger, Fikri M. Abu-Zidan, Edoardo Picetti, Luca Ansaloni, Goran Augustin, Walter L. Biffl, Marco Ceresoli, Osvaldo Chiara, Massimo Chiarugi, Raul Coimbra, Yunfeng Cui, Dimitris Damaskos, Salomone Di Saverio, Joseph M. Galante, Vladimir Khokha, Andrew W. Kirkpatrick, Kenji Inaba, Ari Leppäniemi, Andrey Litvin, Andrew B. Peitzman, Vishal G. Shelat, Michael Sugrue, Matti Tolonen, Sandro Rizoli, Ibrahima Sall, Solomon G. Beka, Isidoro Di Carlo, Richard Ten Broek, Chirika Mircea, Giovanni Tebala, Michele Pisano, Harry van Goor, Ronald V. Maier, Hans Jeekel, Ian Civil, Andreas Hecker, Edward Tan, Kjetil Soreide, Matthew J. Lee, Imtiaz Wani, Luigi Bonavina, Mark A. Malangoni, Kaoru Koike, George C. Velmahos, Gustavo P. Fraga, Andreas Fette, Nicola de’Angelis, Zsolt J. Balogh, Thomas M. Scalea, Gabriele Sganga, Michael D. Kelly, Jim Khan, Philip F. Stahel, Ernest E. Moore

**Affiliations:** 1 Director of Acute Care Surgery and Trauma Unit, Department of General Surgery, Hadassah Medical Center and Faculty of Medicine, Hebrew University of Jerusalem Kiriat Hadassah, POB 12000, 91120 Jerusalem, Israel; 2 General and Emergency Surgery Department, Bufalini Hospital, Cesena, Italy; 3 Tel Aviv Sackler School of Medicine, Tel Aviv, Israel; 4 Department of General, Digestive and Metabolic Minimally Invasive Surgery, Centre Hospitalier Intercommunal De Poissy/St Germain en Laye, Poissy, France; 5 Department of Surgery, Faculdade de Ciências Médicas e da Saúde de Juiz de Fora, Hospital Universitário Terezinha de Jesus, Juiz de Fora, Brazil; 6 Department of General Surgery, Royal Perth Hospital, The University of Western Australia, Perth, Australia; 7 Department of Surgery, Macerata Hospital, Macerata, Italy; 8 Department of General, Emergency and Trauma Surgery, Pisa University Hospital, Pisa, Italy; 9 Department of General Surgery, Rambam Health Care Campus, Haifa, Israel; 10 Department of Surgery, College of Medicine and Health Sciences, United Arab Emirates University, Al-Ain, United Arab Emirates; 11 Department of Anesthesia and Intensive Care, Azienda Ospedaliero-Universitaria Parma, Parma, Italy; 12 Department of Surgery, Fondazione IRCCS Policlinico San Matteo, University of Pavia, Pavia, Italy; 13 Department of Surgery, University Hospital Centre Zagreb, Zagreb, Croatia; 14 Division of Trauma/Acute Care Surgery, Scripps Clinic Medical Group, La Jolla, CA USA; 15 Emergency and General Surgery Department, School of Medicine and Surgery, University of Milano-Bicocca, Monza, Italy; 16 Emergency Department, Niguarda Ca’Granda Hospital, Milan, Italy; 17 CECORC Research Center, Riverside University Health System, Loma Linda University, Loma Linda, USA; 18 Department of Surgery, Nankai Clinical School of Medicine, Tianjin Nankai Hospital, Tianjin Medical University, Tianjin, China; 19 Department of Surgery, Royal Infirmary of Edinburgh, Edinburgh, UK; 20 General Surgery Department Hospital of San Benedetto del Tronto, Marche region, Italy; 21 Division of Trauma and Acute Care Surgery, Department of Surgery, University of California Davis, Sacramento, CA USA; 22 Department of Emergency Surgery, City Hospital, Mozyr, Belarus; 23 General, Acute Care, Abdominal Wall Reconstruction, and Trauma Surgery, Foothills Medical Centre, Calgary, AB Canada; 24 Division of Trauma and Surgical Critical Care, Department of Surgery, University of Southern California, Los Angeles, CA USA; 25 Abdominal Center, Helsinki University Hospital and University of Helsinki, Helsinki, Finland; 26 Department of Surgical Disciplines, Regional Clinical Hospital, Immanuel Kant Baltic Federal University, Kaliningrad, Russia; 27 Department of Surgery, University of Pittsburgh School of Medicine, UPMC-Presbyterian, Pittsburgh, USA; 28 Department of General Surgery, Tan Tock Seng Hospital, Novena, Singapore; 29 Donegal Clinical Research Academy Emergency Surgery Outcome Project, Letterkenny University Hospital, Donegal, Ireland; 30 Surgery Department, Section of Trauma Surgery, Hamad General Hospital (HGH), Doha, Qatar; 31 General Surgery Department, Military Teaching Hospital, Dakar, Senegal; 32 Ethiopian Air Force Hospital, Bishoftu, Ethiopia; 33 Department of Surgical Sciences and Advanced Technologies, General Surgery Cannizzaro Hospital, University of Catania, Catania, Italy; 34 Department of Surgery, Radboud University Medical Center, Nijmegen, The Netherlands; 35 Centre Hospitalier Universitaire Grenoble Alpes, Grenoble, France; 36 Department of Digestive and Emergency Surgery, S.Maria Hospital Trust, Terni, Italy; 37 General and Emergency Surgery, ASST Papa Giovanni XXIII, Bergamo, Italy; 38 Harborview Medical Center, University of Washington School of Medicine, Seattle, WA USA; 39 Department of Surgery, Erasmus University Medical Centre, Rotterdam, The Netherlands; 40 Faculty of Medical and Health Sciences, University of Auckland, Auckland, New Zealand; 41 Emergency Medicine Department of General and Thoracic Surgery, University Hospital of Giessen, Giessen, Germany; 42 HPB Unit, Department of Gastrointestinal Surgery, Stavanger University Hospital, Stavanger, Norway; 43 Department of Oncology and Metabolism, University of Sheffield, Sheffield, UK; 44 Government Gousia Hospital, Srinagar, India; 45 Department of Surgery, IRCCS Policlinico San Donato, University of Milano, Milano, Italy; 46 Case Western Reserve University School of Medicine, Cleveland, USA; 47 Kyoto Medical Center, Kyoto, Japan; 48 Division of Trauma, Emergency Surgery and Surgical Critical Care, Massachusetts General Hospital, Boston, PA USA; 49 Division of Trauma Surgery, School of Medical Sciences, University of Campinas (Unicamp), Campinas, Brazil; 50 Pediatric Surgery, Children’s Care Center, SRH Klinikum Suhl, Suhl, Thueringen Germany; 51 Unit of Digestive and HPB Surgery, Faculty of Medicine, University of Paris, Paris, France; 52 John Hunter Hospital and University of Newcastle, Newcastle, NSW Australia; 53 Cowley Shock Trauma Center at the University of Maryland, Baltimore, MD USA; 54 Emergency Surgery and Trauma, Fondazione Policlinico Universitario A. Gemelli IRCCS, Università Cattolica del Sacro Cuore, Rome, Italy; 55 Department of General Surgery, Albury Hospital, Albury, Australia; 56 University of Portsmouth, Portsmouth Hospitals University NHS Trust, Portsmouth, UK; 57 College of Osteopathic Medicine, Rocky Vista University, Parker, CO USA; 58 Ernest E Moore Shock Trauma Center at Denver Health, University of Colorado, Denver, CO USA

**Keywords:** Mesenteric ischemia, Mesenteric arterial occlusion, Mesenteric artery stenting, Bowel ischemia, Guidelines, Recommendations, World Society of Emergency Surgery

## Abstract

Acute mesenteric ischemia (AMI) is a group of diseases characterized by an interruption of the blood supply to varying portions of the intestine, leading to ischemia and secondary inflammatory changes. If untreated, this process may progress to life-threatening intestinal necrosis. The incidence is low, estimated at 0.09–0.2% of all acute surgical admissions, but increases with age. Although the entity is an uncommon cause of abdominal pain, diligence is required because if untreated, mortality remains in the range of 50%. Early diagnosis and timely surgical intervention are the cornerstones of modern treatment to reduce the high mortality associated with this entity. The advent of endovascular approaches in parallel with modern imaging techniques is evolving and provides new treatment options. Lastly, a focused multidisciplinary approach based on early diagnosis and individualized treatment is essential. Thus, we believe that updated guidelines from World Society of Emergency Surgery are warranted, in order to provide the most recent and practical recommendations for diagnosis and treatment of AMI.

**Supplementary Information:**

The online version contains supplementary material available at 10.1186/s13017-022-00443-x.

## Background

Acute mesenteric ischemia (AMI) is caused by sudden interruption of blood supply to the intestine, leading to cellular damage, intestinal necrosis, and commonly patient death if untreated [1]. AMI may be occlusive or non-occlusive (NOMI), with the primary etiology further defined as mesenteric arterial embolism (50%), mesenteric arterial thrombosis (15–25%), or mesenteric venous thrombosis (5–15%) [2, 3]. The overall incidence is low (0.09–0.2% of all acute admissions to emergency departments), representing an infrequent cause of abdominal pain [4–6], but a common cause of emergent intestinal resection. Prompt diagnosis and intervention are essential to reduce the mortality rates that exceed 50% [7–10].

Traditionally, AMI has been treated with open surgery. Over the past two decades, the rapid development of endovascular techniques has made this approach an important alternative for patients with occlusion of the superior mesenteric artery (SMA). Some studies have shown that endovascular therapy is associated with lower rates of mortality and bowel resection than the traditional, open approach [11–13].

The assessment and therapy carried out by an interdisciplinary team should keep the time-to-reperfusion interval as short as possible. In addition, advances in postoperative care have improved outcome for patients with short bowel syndrome [14, 15]. Both in-hospital care and further bowel rehabilitation lead to increase survival and better long-term outcome with acceptable quality of life [16, 17].

Introducing a clinical pathway and centers of excellence results in higher awareness of AMI, more appropriate imaging, less delays, increased number of revascularizations, and, therefore, lower mortality [18, 19].

Accordingly, the present paper aims to provide an update with recommendations based on the most currently accepted concepts in the management of AMI [20].

## Methods

The World Society of Emergency Surgery (WSES) endorsed a team of experts to develop specific questions about diagnosis and management of AMI. This group performed a thorough literature review and presented its findings during the WSES World Congress, September 2021 in Edinburg, Scotland. The quality of the evidence available was evaluated according to the GRADE methodology, and recommendations were classified into two levels: strong recommendation in favor or against; weak recommendation (suggestion) in favor or against. [21–24]

During the Congress, the Board of the Society approved the proposed statements. After the acceptance, the update of the guidelines was further discussed by the Board of the WSES and approved.

## Pathophysiology and epidemiology

### Acute mesenteric arterial embolism

Half of cases of AMI are due to acute SMA embolism [2, 3]. Mesenteric emboli can originate from the left atrium (e.g., atrial fibrillation), left ventricle (e.g., left ventricular dysfunction with poor ejection fraction), or cardiac valves (e.g., endocarditis). Occasionally emboli are generated from an atherosclerotic aorta. Emboli typically lodge at points of normal anatomic artery narrowing. The SMA is particularly vulnerable because of its relatively large diameter and low takeoff angle from the aorta. The majority of emboli lodge 3–10 cm distal to the origin of the SMA, thus sparing the proximal jejunum and colon. More than 20% of SMA emboli are associated with concurrent emboli to another arterial bed including the spleen and kidney [25].

### Acute mesenteric arterial thrombosis

Thrombosis of the SMA (approximately 25% of cases) is usually associated with pre-existing chronic atherosclerotic disease leading to stenosis. Many of these patients have a history consistent with chronic mesenteric ischemia (CMI), including postprandial pain, weight loss, or “food fear.” A detailed medical history is important when evaluating a patient suspected to have AMI. Thrombosis usually occurs at the origin of visceral arteries. An underlying plaque in the SMA usually progresses eventually to a critical stenosis resulting in collateral beds. Accordingly, symptomatic SMA thrombosis most often accompanies celiac occlusion [26]. SMA thrombosis may also occur due to vasculitis, mesenteric dissection, or mycotic aneurysm. Involvement of the ileocolic artery will result in necrosis of the proximal colon.

### Acute non-occlusive mesenteric ischemia

NOMI occurs in approximately 20% of cases, and is usually a consequence of SMA vasoconstriction associated with low splanchnic blood flow [27]. The compromised SMA blood flow also affects the proximal colon due to involvement of the ileocolic artery. Patients with NOMI typically suffer from severe coexisting illness, commonly cardiac failure which may be precipitated by sepsis. Hypovolemia and the use of vasoconstrictive agents may precipitate NOMI.

### Mesenteric venous thrombosis

Mesenteric venous thrombosis (MVT) accounts for less than 10% of cases of mesenteric infarction. Thrombosis is attributed to a combination of Virchow’s triad; stagnant blood flow, hypercoagulability, and endothelial damage. In young patients, 36% of MVT occurs without an obvious cause [28]. An inflammatory process around the superior mesenteric vein (SMV) due to acute pancreatitis or inflammatory bowel disease may cause thrombosis. Surgical trauma such as splenectomy or bariatric surgery may also provoke SMV thrombosis. Hypercoagulability may be due to inherited disease such as Factor V Leiden, prothrombin mutation, protein S deficiency, protein C deficiency, antithrombin deficiency, and antiphospholipid syndrome. Additionally, recent work suggests that fibrinolysis shutdown (resistance to tissue plasminogen activator—tPA) is a significant risk factor for hypercoagulability [29]. Thrombophilia may also be acquired due to malignancies, hematologic disorders, and oral contraceptives [30].

### Recent trends: prevalence, pathophysiology

The prevalence of AMI has changed in recent decades. The prevalence of acute mesenteric occlusion among patients with an acute abdomen may vary from 17.7% in emergency laparotomy and 31.0% in laparotomy for elderly non-trauma patients [31].

Mesenteric arterial embolism decreased to 25% of cases [3, 32]. Mesenteric arterial thrombosis was the second most common cause of mesenteric ischemia, which historically accounted for 20–35% and recently increased to 40% [32]. NOMI accounts for 25% of cases [3], which is also increasing, compared to the historical cohort, because of increased number of critically ill patients and overall improvement of intensive care. Although the mechanism is still unknown, heart failure, renal failure, cardiac surgery using cardiopulmonary bypass, and the use of catecholamine are reported as risk factors [33].

The etiology of AMI has changed over the years with increasing percentages of acute arterial thrombosis due to atherosclerosis which may in part be explained by modern anticoagulant therapy used for the treatment of atrial fibrillation.

The incidence of AMI increases exponentially with age. In patients aged 75 years or older, AMI is a more prevalent cause of acute abdomen than appendicitis [1]. The incidence of AMI in an 80-year-old is roughly tenfold that of a 60-year-old patient [34].

Abdominal compartment syndrome with very high intraabdominal pressure may cause bowel ischemia that is complicated with ischemia–reperfusion injury when decompression laparotomy is performed [35].

AMI has been described in patients with coronavirus disease (COVID -19), probably related to large vessel thromboembolic events as well as to small vessel thrombosis linked to hypercoagulability and fibrinolysis shutdown [36].

1. Severe abdominal pain out of proportion to physical examination findings should be assumed to be AMI until disproven. (Strong recommendation based on low-quality evidence 1C)

The key to early diagnosis is a high level of clinical suspicion.

The clinical scenario of a patient complaining of excruciating abdominal pain with an unrevealing abdominal examination is classic for early AMI [37]. The reason for the pain being disproportionate to the clinical findings is that ischemia starts from the mucosa toward the serosa. That is why initially there is severe pain without clinical findings.

If the physical examination demonstrates signs of peritonitis, there is likely irreversible intestinal ischemia with bowel necrosis. In a study on AMI, 95% of patients presented with abdominal pain, 44% with nausea, 35% with vomiting, 35% with diarrhea, and 16% with blood per rectum [38]. Approximately, one-third of patients present with the triad of abdominal pain, fever, and hemoccult-positive stool. Other patients, particularly those with delayed diagnosis, may present in extremis with septic shock. Clinical signs of peritonitis may be subtle. Accordingly, one must have a high index of suspicion, because such findings are predictive of intestinal infarction.

The classic presentation of AMI, i.e., “severe, poorly localized abdominal pain that is out of proportion to the physical examination,” is becoming less common, while the “acute on chronic” presentations of mesenteric ischemia are more typical, and probably underdiagnosed [39]. Patients presenting with symptomatic chronic mesenteric ischemia are at high risk of developing in-hospital AMI.

Severe COVID-19 infection and AMI have a poor prognosis, delay in diagnosis, and intervention [40–42]. AMI should be suspected in patients with COVID-19 who present with nausea, vomiting, diarrhea, abdominal pain, and abdominal distension because of hypercoagulability and hypoperfusion. Blood tests will not aid in the diagnosis of AMI, though essential in patient management. CTA is the diagnostic modality of AMI along with clinical correlation.

2. Clinical scenario and risk factors differentiate AMI as mesenteric arterial emboli, mesenteric arterial thrombosis, NOMI, or mesenteric venous thrombosis. (Weak recommendation based on low-quality evidence 1C)

#### Types of AMI

A careful medical history is important because distinct clinical scenarios are associated with the pathophysiological form of AMI [43]. Patients with mesenteric arterial thrombosis often have a history of chronic postprandial abdominal pain, progressive weight loss, and previous revascularization procedures for mesenteric arterial occlusion. Patients with NOMI have pain that is generally more diffuse and episodic associated with poor cardiac performance. These patients are more likely to have suffered from cardiac failure, and recent surgery. Several other smaller cohorts also reported hemodialysis as a risk factor of NOMI [44, 45]. Furthermore, NOMI represents a cause of secondary worsening in septic shock, particularly in septic patients treated with high-dose vasoactive drugs.

Patients with MVT present with a mixture of nausea, vomiting, diarrhea, and abdominal cramping. Gastrointestinal bleeding occurs in 10% [46].

Nearly 50% of patients presenting with embolic AMI have atrial fibrillation, and approximately one-third of patients have a prior history of arterial embolus with preexisted peripheral vascular disease [38].

Risk factors for specific phenotypes of AMI are presented in Table 1.^.^

3. Plain X-ray is not recommended in evaluating patients for intestinal ischemia. (Strong recommendation based on moderate-quality evidence 1B)

**Table 1:** Risk factors for specific types of AMI

	Pathogenesis of AMI			
	Acute mesenteric arterial embolism	Acute mesenteric arterial thrombosis	NOMI	Mesenteric venous thrombosis
Risk factors	Atrial fibrillation recent MI cardiac thrombi Mitral valve disease Left ventricular aneurysm Endocarditis Previous embolic disease	Diffuse atherosclerotic disease Postprandial pain Weight loss	Cardiac failure Low flow states Multiorgan dysfunction Vasopressors Abdominal compartment syndrome	Portal hypertension history of VTE Oral Contraceptives Estrogen use Thrombophilia Pancreatitis
Clinical onset	Sudden strong abdominal pain, vomiting	Progressive or sudden abdominal pain, vomiting, diarrhea and/or melena	Progressive pain, mild	Nonspecific GI symptoms, abdominal distension, worsening of general condition
Vascular involvement	Main artery or branches of SMA	Celiac trunk, SMA, IMA origins	Superior mesenteric vein, progression to portal vein	Stenosis of SMA

*AMI* Acute mesenteric ischemia; *NOMI* Non-occlusive mesenteric ischemia; *MI* Myocardial infarction; *SMA* Superior mesenteric artery; *IMA* Inferior mesenteric artery; *GI* Gastrointestinal; and *VTE* Venous thromboembolism

A radiograph is usually the initial test ordered in patients with acute abdominal pain but has a limited role in the diagnosis of mesenteric ischemia, especially in the early setting. A negative radiograph does not exclude mesenteric ischemia [47]. Plain radiography only becomes positive when bowel infarction has developed and intestinal perforation manifests as free intraperitoneal air.

4. There are no laboratory parameters that are sufficiently accurate to conclusively identify the presence or absence of ischemic or necrotic bowel, although elevated l-lactate, leukocytosis, and D-dimer may assist. (Weak recommendation based on moderate-quality evidence 2B)

Although laboratory results are not definitive, they may help to corroborate clinical suspicion. More than 90% of patients will have an abnormally elevated leukocyte count [48]. The second most commonly encountered abnormal finding is metabolic acidosis with elevated lactate level, which occurs in 88% [49].

Patients may present with lactic acidosis due to dehydration and decreased oral intake. Thus, differentiation of early ischemia versus irreversible bowel injury based upon the lactate level alone is not reliable unless accompanied by other clinical evidence. Elevated serum lactate levels > 2 mmol/l is associated with irreversible intestinal ischemia hazard ratio: 4.1 (95% CI: 1.4–11.5; *p* < 0.01) in case of AMI [50].

It should be emphasized that the presence of lactic acidosis in combination with abdominal pain when the patient may not otherwise appear clinically ill should lead to consideration of early CTA.

Based on the current literature, no accurate biomarkers have been identified to diagnose AMI [51, 52]. D-dimer has been reported to be an independent risk factor for intestinal ischemia [52], reflecting ongoing clot formation and endogenous degradation via fibrinolysis. No patient presenting with a normal D-dimer had intestinal ischemia and D-dimer > 0.9 mg/L had a specificity, sensitivity, and accuracy of 82%, 60%, and 79%, respectively [53]. Thus, D-dimer may be useful in the early assessment.

Elevated amylase has been reported in roughly a half of patients with AMI. [54] This is important to note to as patient may be misdiagnosed as having acute pancreatitis, and delay in critical interventions could impact survival outcomes.

Other biomarkers reported to be of use in the diagnosis of AMI include intestinal fatty acid-binding protein (I-FABP), serum alpha-glutathione S-transferase (alpha-GST), and cobalt–albumin binding assay (CABA) [55, 56]. A cross-sectional diagnostic study of 129 patients admitted for acute abdominal pain found that the three most promising circulating biomarkers for AMI—citrulline, I-FABP, and *d*-lactate—were neither sensitive nor specific enough for the differential diagnosis of AMI [57].

These results, however, contrast with other published reports [56, 58]. This could be explained by selection bias (established severe AMI cases were included) leading to an overestimated performance of the studied biomarkers.

5. Computed tomography angiography (CTA) should be performed without delay in any patient with suspicion for AMI. (Strong recommendation based on high-quality evidence 1A)

Delay in diagnosis is the dominant factor that accounts for high mortality rates of 30–70% despite increased knowledge of this entity [59, 60]. Every 6 h of delay in diagnosis (actually—delay in CTA) doubles mortality [61]. The multidetector CTA has replaced formal angiography as the diagnostic study of choice. Volume rendering is now a semiautomatic workflow component of many CT machines. These can aid remote communities with less experienced staff.

In the presence of advanced AMI, the CTA findings reflect irreversible ischemia (intestinal dilatation and thickness, reduction or absence of visceral enhancement, pneumatosis intestinalis, and portal venous gas, especially the combination of all) and free intraperitoneal air [62].

Comprehensive biphasic CTA includes the following important steps:

- Pre-contrast scans to detect vascular calcification, hyper-attenuating intravascular thrombus, and intramural hemorrhage.
- Arterial and venous phases to demonstrate thrombus in the mesenteric arteries and veins, abnormal enhancement of the bowel wall, and the presence of embolism or infarction of other organs.
- Multiplanar reconstructions (MPR) to assess the origin of the mesenteric arteries [63].

The oral contrast is not indicated and even harmful. CTA should be performed despite the presence of acute kidney injury, as the consequences of delayed or missed diagnosis are far more detrimental for patients than exposure to the iodinated contrast agent. A recent study found that in 27 of 28 patients (96.4%) MDCT correctly diagnosed AMI (specificity of 97.9%) [27, 64]. A sensitivity of 93%, specificity of 100%, and positive and negative predictive values of 100% and 94%, respectively, were achieved [65, 66].

Six radiological findings (bowel loop dilatation, pneumatosis intestinalis, SMV thrombosis, free intraperitoneal fluid, portal vein thrombosis, and splenic vein thrombosis) were found to be predictors of bowel necrosis in patients with AMI [67]. The clinical significance of pneumatosis intestinalis as a single radiological finding remains the challenge. In a biggest multicentral retrospective study, 60% of patients had benign disease [68].

In NOMI, CTA may demonstrate bowel ischemia and free fluid in the face of patent mesenteric vessels. In MVT, the most common positive radiological finding on venous phase CTA is thrombus in the superior mesenteric vein described as the target sign [69].

Associated findings that suggest MVT include bowel wall thickening, pneumatosis, splenomegaly, and ascites [69]. Portal or mesenteric venous gas strongly suggests the presence of bowel infarction.

Diagnostic angiography can differentiate occlusive, embolic, and thrombotic from non-occlusive AMI.

Duplex ultrasonography has a limited role in this entity, but may be helpful if obtained early in chronic cases [47]. It could be useful to monitor the bowel’s peristalsis or the amount of free peritoneal fluid especially in NOMI.

MRA is an established technique in the evaluation of the mesenteric arterial and venous vasculature in patients with suspected AMI. It has been well accepted for chronic mesenteric ischemia cases and functional assessment of bowel insufficiency as a result of SMA pathology [70]. Nevertheless, its use is limited in the emergency setting.

6. Non-occlusive mesenteric ischemia (NOMI) should be suspected in critically ill patients with abdominal pain or distension requiring vasopressor support and evidence of multiorgan dysfunction. (Weak recommendation based on low-quality evidence 2C)

Clinical examination and routine laboratory tests are of only little value in reaching an early and reliable diagnosis of NOMI. Unexplained abdominal distension or gastrointestinal bleeding may be the only signs of acute intestinal ischemia in NOMI and may be undetectable in sedated patients in the ICU in approximately 25% of cases [71, 72]. Patients surviving cardiopulmonary resuscitation who develop bacteremia and diarrhea should be suspected of having NOMI, regardless of presence or absence of abdominal pain. Right-sided abdominal pain associated with the passage of maroon or bright red blood in the stool is highly suggestive of NOMI.

Gastrointestinal perfusion is often impaired early in critical illnesses, major surgery, or trauma, all of which are characterized by increased demands on the circulation to maintain tissue oxygen delivery [73].

Most of the symptoms listed in this section are often not clinically apparent in a critically ill and ventilated patients. Accordingly, any negative changes in a patient's physiology, including new onset of organ failure, increase in vasoactive support, and nutrition intolerance, should raise the suspicion of AMI.

Experimental and observational studies suggest that the use of vasopressors such as norepinephrine and epinephrine might result in impaired mucosal perfusion [74, 75]. Other pharmacological agents such as vasopressin and digoxin [76] as well as acute profound hypovolemia could also worsen ischemia.

Lastly, the role of enteral nutrition in critically ill patients on development of intestinal ischemia is controversial. In general, enteral and parenteral nutrition is complementary to meet patient’s daily caloric requirements. In the recent randomized controlled trial “NUTRIREA 2” [77], enteral nutrition was compared to parenteral nutrition: Mortality did not differ between the two groups, but a significantly higher rate of bowel ischemia was reported in the enteral group.

7. When the diagnosis of AMI is made, fluid resuscitation should commence immediately to enhance visceral perfusion. Electrolyte abnormalities should be corrected, and nasogastric decompression initiated. (Strong recommendation based on moderate-quality evidence 1B)

Fluid resuscitation with crystalloid and blood products is essential for the management of the patient with suspected AMI. Preoperative resuscitation is important to prevent cardiovascular collapse on induction of anesthesia. To guide effective resuscitation, early hemodynamic monitoring should be implemented [78]. Assessment of electrolyte levels and acid–base status should be performed. This is especially true in patients with AMI, where severe metabolic acidosis and hyperkalemia may result from underlying bowel infarction and reperfusion [79]. Vasopressors should be used with caution. Dobutamine, low-dose dopamine, and milrinone to improve cardiac function have been shown to have less impact on mesenteric blood flow [80, 81]. The fluid volume requirement in these patients may be high, due to extensive capillary leakage, but the infusion of large volume of crystalloid should be utilized carefully to optimize bowel perfusion [82]. The goals of therapy should address physiologic levels of oxygen delivery with continued monitoring of lactate level as an indication of perfusion improvement. Supra-physiologic level of oxygen delivery was suggested in the past which is not supported by the current evidence [83].

8. Broad-spectrum antibiotics should be immediately administered. (Strong recommendation based on moderate-quality evidence 1C)

The high risk of infection among patients with AMI outweighs the risks of acquired antibiotic resistance, and therefore, broad-spectrum antibiotics should be administered early in the course of treatment [84]. Intestinal ischemia leads to early loss of the mucosal barrier, which facilitates bacterial translocation and the risk of septic complications. Antibiotic therapy should be administered for at least 4 days in immunocompetent stable patients with consideration given to a longer duration of therapy for signs of ongoing infection [85]. As soon as possible, antibiotic regimen should be tailored according to the microbial isolation. Prolonged course of empiric antibiotics, if clinically deemed necessary, should be guided in accordance with local antibiotic stewardship team.

9. Prompt laparoscopy/laparotomy should be done for patients with an overt peritonitis. (Strong recommendation based on low-quality evidence 1C)

When physical findings suggestive of an acute intraabdominal catastrophe are present, bowel infarction has already occurred, and the chance of survival in this patient population with significant associated comorbidity is reduced dramatically. Peritonitis secondary to bowel necrosis mandates surgery without delay.

The goal of surgical intervention for AMI includes:

1. Re-establishment of the blood supply to the ischemic bowel.
2. Resection of all non-viable regions.
3. Preservation of all viable bowel.

Intestinal viability is the most important factor influencing outcome in patients with AMI. Non-viable intestine, if unrecognized, results in multisystem organ dysfunction and ultimately death. Prompt laparotomy allows for direct assessment of bowel viability.

#### Emergency laparotomy

After initial resuscitation, midline laparotomy should be performed, followed by the assessment of all areas of the intestine with decisions for resection of all frankly necrotic areas. The SMA is easily palpated by placing fingers behind the root of the mesentery. The SMA is identified as a firm tubular structure, which may have or not a palpable pulse. Otherwise, the SMA can also be reached by following the middle colic artery where it enters the SMA at the mesentery. Direct sharp dissection, exposing the artery from its surrounding mesenteric tissue, is required for proper exposure to perform revascularization. In cases where there is diagnostic uncertainty, arteriogram is the study of choice. It can be done intraoperatively especially in hybrid suites. Intraoperative duplex is a simple, rapid, repeatable, and often definitive alternative diagnostic modality.

#### Re-establishment of the blood supply to the ischemic bowel

Revascularization when relevant has an essential role in the multidisciplinary approach to AMI. As an example, among the 104 patients who did not undergo revascularization, 64 (62%) died within 30 days compared to 36 out of 85 (42%) patients who were re-vascularized (*p* = 0.01) [86].

Different techniques of blood flow restoration are used depending on the AMI pathophysiology. Embolectomy and angioplasty are a well-established definitive treatment for SMA emboli. On the other hand, thrombosis of the SMA at the origin of the aorta (a common pathology in diffuse atherosclerosis) will require a bypass procedure. Bypass may be performed in either an antegrade fashion from the supraceliac aorta or retrograde fashion from the infrarenal aorta or common iliac arteries. Single-vessel revascularization (SMA) is usually sufficient in the acute setting. However, it increases the magnitude of the procedure and may require prosthetics in the presence of contaminated field. Nowadays endovascular procedures reduce the requirement for surgical bypasses. Thus, multidisciplinary collaborative approach including specialists from multiple disciplines is integral for good clinical outcomes.

Temporary SMA shunting may spare considerable bowel. For patients in extremis, or where the necessary technical skillset is not available, temporary SMA shunting should be considered.

Neither NOMI nor MVT typically requires vascular repair. Full-dose anticoagulation should be initiated on all patients prior to the surgical procedure. Unfractionated heparin is effective and easy to manage, especially in patients with acute kidney failure.

#### Intraoperative bowel viability assessment

There are limited intraoperative tools to help surgeons in decision making regarding bowel viability, especially in circumstances in which the bowel appears to be “dusky” or threatened but not clearly ischemic. In this case, a temporary abdominal closure via a negative pressure wound therapy device or temporary dressing (custom made with plastic sheets, gauzes, and drains) is convenient in order to provide an opportunity for a second-look surgery. Clear documentation of bowel length is crucial in every operation note.

In addition to traditional surgical inspection of the bowel, available techniques of intraoperative assessment of bowel viability rely on bowel oxygenation, myoelectric activity, and perfusion. The intraoperative absence of any one of these criteria is a sufficient predictor of bowel non-viability.

Many surgeons use their hands and eyes to look for the presence or absence of peristalsis or mesenteric pulsation to evaluate whether blood flow is adequate.

Doppler ultrasonography (DUS) is a safe and noninvasive technique to measure blood flow and is popular for its easiness of use and relatively low cost [87].

Flowmetry with fluorescein dye is currently part of the accepted standard of clinical care for intraoperative assessment of bowel viability. Fluorescein can therefore be used to visualize perfusion in open laparotomies using a Woods Lamp or laparoscopically using an endoscope with appropriate filters [88, 89].

Indocyanine Green (ICG) is a near-infrared (NIR) fluorophore with an emission peak of 832 nm in whole blood [90]. It has been used in the same way as fluorescein, but primarily in the elective surgical setting [91]. ICG utilization in the emergent setting, particularly in AMI, has not been well investigated to date, although early animal models, isolated cases, and cohort studies show promise.

The potential for combining modalities for intraoperative bowel assessment warrants further studies [92].

#### Laparoscopy in AMI

Diagnostic laparoscopy is feasible as a bedside procedure in the intensive care unit (ICU) with the advantage of avoiding time delay for awaiting operating room availability and preventing adverse events during critically ill patients transfer. However, the routine use of diagnostic laparoscopy in AMI has not been generally adopted [6].

When a second-look surgery is indicated, second-look laparoscopy may be a useful alternative to conventional surgery, because it prevents critically ill patients from the trauma and risks of relaparotomy and can be performed as an ICU bedside operation. In one study, only 20% of patients underwent a second-look laparoscopy within first 72 h, but this did not change the outcome and complication rate [93]. In another case series, non-therapeutic laparotomy was avoided in 9/20 patients with NOMI [94].

The European Association for Endoscopic Surgery (EAES) consensus for the laparoscopic approach to the acute abdomen states that there is no published data demonstrating advantages in the diagnosis and treatment of acute bowel ischemia by laparoscopy [95]. However, laparoscopy can be useful in confirming the diagnosis in doubtful cases, evaluate the extension of the ischemic small bowel segment, and offer a treatment option in cases of segmental necrosis.

In addition, post-cardiac surgery patients admitted to the ICU have a relatively high rate of NOMI, where the CT-scan can be equivocal. In these cases, bedside diagnostic laparoscopy may be a safe and effective procedure that avoids needless laparotomy and can direct further management steps [96].

10. Endovascular revascularization procedures are the primary option in cases of arterial occlusion when sufficient expertise is available. (Strong recommendation based on low-quality evidence 1C)

Endovascular techniques have become popular in revascularization of the SMA. No randomized control trial has been performed to assess and compare open surgery to an endovascular approach, as patients with AMI are very heterogenic and physiologically different [97]. Much controversy surrounds the use of endovascular techniques as primary management of AMI [98]. Some studies report lesser need for laparotomy, less bowel resection, and significantly lower mortality rate with endovascular techniques compared to surgery [99].

Open surgery is effective in assessing the viability of the bowel and hence preventing delay in revascularization especially when an endovascular approach is unavailable [1].

Different endovascular procedures are summarized in Table 2.

**Table 2:** Endovascular procedures in occlusion of SMA

Endovascular procedure	Advantages and challenges	References
Aspiration embolectomy	Lower mortality Patients without peritonitis Repeat procedures	[100, 101]
SMA thrombolysis	Bleeding complications Laparotomy required in 38% Patients without peritonitis Contraindicated: recent surgery, trauma, cerebrovascular or gastrointestinal bleeding, and uncontrolled hypertension	[102]
Antegrade stenting	Risk of dissection Unfavorable artery angulation	[103]
Retrograde stenting	Laparotomy necessary Avoiding bypass when necrotic bowel presents Success 94%	[104–106]

#### Comparison of endovascular intervention and surgery

Publications related to endovascular treatment of AMI have been evolving since 2010 [11, 12]. Several observational studies and meta-analyses comparing the outcomes of endovascular interventions and surgery have been published [13, 107–110].

All studies have shown a benefit for endovascular therapy compared to open surgery in terms of lower bowel resection rates and lower 30-day mortality rates.

The latest study using the National Inpatient Sample database included 4665 patients who underwent interventional treatment (24% endovascular and 76% open revascularization) from 2005 through 2009 showed that endovascular intervention is associated with lower mortality compared to open surgery (24.9% vs 39.3%) [111]. Another meta-analysis including nineteen observational studies also showed that endovascular intervention was associated with a lower prevalence of bowel resection (OR 0.45, 95%CI 0.34–0.59) and 30-day mortality (OR 0.45, 95%CI 0.34–0.59) compared with open surgery [112].

The Guidelines of the European Society of Vascular Surgery showed a pooled overall 30-day mortality rate after endovascular therapy of 17.2% (367/2131), compared to 38.5% after open surgery (1582/4111) [113].

It is important to note that all studies that were focused on endovascular revascularization have high levels of heterogeneity. It is possible that patients undergoing open repair have more advanced disease resulting in long-segment bowel resection rates and poorer outcome. The 5-year survival following endovascular treatment and open vascular surgery was 40% and 30%, respectively [108].

The pooled estimate of technical success of endovascular intervention was 94%, based on a recent meta-analysis [100]. On the other hand, the pooled estimate of the unplanned surgery rate of endovascular therapy was 40%.

In patients with acute embolic SMA occlusion, there are no data suggesting a superiority of open versus endovascular treatment [114].

Aspiration embolectomy with thrombolytic treatment should be considered in patients with no clinical signs of acute peritonitis. In a study that analyzed the Swedish Vascular Registry (SWEDVASC) between 1987 and 2009, 34 patients that received thrombolysis for acute SMA occlusion were identified. In-hospital mortality was 26%, and technical success was 88%. Patients who needed explorative laparotomy after lysis had an in-hospital mortality rate of 38% [102].

#### Hybrid approach: endovascular intervention and surgery

Retrograde open mesenteric stenting (ROMS) is an emerging hybrid technique utilized in cases of AMI. This procedure includes a laparotomy and retrograde endovascular revascularization of the superior mesenteric artery [115]. One of the advantages of this method over vascular bypass is significantly shorter operative time. One of the major concerns after stenting is patency. However, patency rates similar to bypass were reported (76–88%) [116].

Theoretically, if technical capabilities and infrastructure for hybrid procedures are available, ROMS may be a good treatment option for patients who require laparotomies. It is possible that ROMS may avoid the need for second-look surgeries.

Centers of excellence equipped with hybrid operating rooms may provide further data supporting the use of an endovascular strategy [117]. The raised awareness of AMI, low threshold for suspicion, immediate CTA with real-time radiology report, and early involvement of senior staff members are increase rapid access and utilization of hybrid operating rooms.

11. Damage control surgery (DCS) with temporary abdominal closure is an important adjunct for patients who require intestinal resection allowing reassessment of bowel viability and in situations of severe abdominal sepsis. (Strong recommendation based on low-quality evidence 1B)

The damage control laparotomy strategy (abbreviated laparotomy) is an accepted technique in trauma care for the past 30 years. It is an important option in the patient with AMI [118]. Damage control is the surgical modality of choice in the critically ill patient with AMI for physiological and technical reasons. The decision to utilize DCS should be made early based upon the response to resuscitation [119]. Advanced age is not a contraindication to DCS as good outcomes have been observed in the elderly [120].

Planned second-look techniques are required after restoration of SMA flow, with or without resection of ischemic bowel (and no anastomosis or stoma) following resuscitation in the ICU [120, 121]. If there is an uncertainty regarding bowel viability, the stapled off bowel ends should be left in discontinuity and re-inspected after a period of continued ICU resuscitation to restore physiological balance. Often, bowel which is borderline ischemic at the initial exploration will improve after restoration of blood supply and physiologic stabilization. Multiple adjuncts have been suggested to assess intestinal viability, but none have proven to be uniformly reliable [122, 123].

Most often, re-exploration should be accomplished within 24–48 h and decisions regarding anastomosis, stoma, or additional resection can be made with plans for sequential abdominal closure.

In a review of 43 patients undergoing open mesenteric revascularization, the authors noted that 11 of the 23 patients undergoing a second-look operation required bowel resection [32]. The bowel in these patients is often very swollen and poses a high risk for anastomotic leak. Recent studies suggest that careful hand sewn techniques are preferable to staples use in this group [124, 125].

These patients often suffer from acidosis, hypothermia, and coagulopathy, which require prompt and ongoing correction. Physiologic restoration is multifactorial and includes careful and limited crystalloid infusion to avoid abdominal compartment syndrome, frequent monitoring of lactate clearance and central venous oxygen saturation, and the use of viscoelastic techniques (TEG, ROTEM) to assess coagulation status and guide ongoing blood product administration. Recent evidence suggests that direct peritoneal resuscitation techniques can be useful in this scenario [126, 127].

12. Mesenteric venous thrombosis can often be successfully treated with a continuous infusion of unfractionated heparin. (Strong recommendation based on moderate-quality evidence 1B)

MVT has a distinctive clinical finding on CTA scan, and when noted in a patient without findings of peritonitis, non-operative management should be considered. The first line treatment for mesenteric venous thrombosis is anticoagulation. Systemic thrombolytic therapy is rarely indicated. When clinical signs demand operative intervention, one should resect only obviously necrotic bowel utilizing damage control techniques since anticoagulation therapy may improve the clinical picture over the ensuing 24–48 h. Early use of heparin has been associated with improved survival [128].

Patients with peritonitis require emergency surgery. Intraoperative management is dictated by the surgical findings ranging from a segmental infarction of small bowel to necrosis of the entire bowel, with or without perforation. The aim of resection is to conserve as much bowel as possible. Second-look laparotomy, 24–48 h later, may avoid the resection of potentially viable bowel. A second-look procedure is mandatory in patients who have extensive bowel involvement.

There are no high-quality studies suggesting that endovascular therapy has a proven role in the treatment of MVT but may be an option in selected patients not responding to anticoagulation therapy. Most published data on interventional radiological treatments for MVT refer to small case series. The use of systemic intravenous tPA has been successfully reported [129]. Thrombolysis via the SMA is ineffective and associated with an increased risk of bleeding [130]. The role of open surgical thrombectomy in modern practice is uncertain [131].

Supportive measures include nasogastric suction, fluid resuscitation, and bowel rest.

13. When NOMI is suspected, the focus is to correct the underlying cause and improve mesenteric perfusion. Infarcted bowel should be resected promptly. (Strong recommendation based on low-quality evidence 1C).

The central principle of NOMI management is the treatment of the underlying precipitating cause. Fluid resuscitation, optimization of cardiac output, and elimination of vasopressors remain important primary measures. Additional treatment may include systemic anticoagulation (heparin) and the use of catheter-directed infusion of vasodilatory and antispasmodic agents, most commonly papaverine hydrochloride [132]. The decision to intervene surgically is based on the presence of peritonitis, perforation, or overall worsening of the patient’s condition [81].

If a patient presents with peritoneal signs, an exploratory laparotomy is required for resection of frankly necrotic bowel. Unfortunately, these patients are often in critical condition and the mortality remains very high (50–85%) [9]. Damage control surgery is an important adjunct, given the critical state of these patients.

Direct vasodilator treatment is not commonly used in real-world practice. Despite several clinical guidelines mentioning vasodilator therapy for NOMI [133–135], only a few small studies have been published [136, 137]. Direct vasodilator infusion of papaverine into the SMA showed reduced mortality associated with AMI [138]. Another study demonstrated that early treatment with continuous IV prostaglandin E1 (PGE1) reduced mortality in patients with NOMI [136].

A nationwide study from Japan focused on vasodilator therapy using papaverine, and/or PGE1 in NOMI patients (161 patients vs. 1676 in control group) showed vasodilator therapy was associated with significantly lower in-hospital mortality and need for abdominal surgery [139]. This was a highly selected patient cohort with mild disease.

14. Postoperative intensive care of AMI patients is directed toward the improved intestinal perfusion and the prevention of a multiple organ failure. (Strong recommendation based on low-quality evidence 1C)

Release of toxic products following bowel resection and restoration of blood flow induce inflammatory processes that can lead to multiorgan failure (MOF) even in the absence of necrotic bowel. Capillary leakage resulting from reperfusion injury leads to volume sequestration into the third space. Systemic hypotension often requires catecholamine administration.

In such a scenario, depending on cardiac output and peripheral vascular resistance, a combination of noradrenaline and dobutamine rather than vasopressin should be considered to minimize the possible negative impact on the intestinal microcirculation [140]. Renal replacement therapy, which is often required in case of acute kidney injury, may contribute to hemodynamic stabilization and facilitate optimization of fluid balance. Because of the potential bacterial translocation from the injured gut, broad-spectrum antibacterial treatment according to current guidelines should be continued after surgery based upon the degree of contamination and culture results [141]. Systemic heparin is administered postoperatively (with activated partial thromboplastin time (aPTT) between 40 and 60) in all patients. Low-molecular weight heparin (LMWH) in therapeutic doses is a good alternative if no surgical interventions are planned. Enteral feeding is preferred, but some patients may need parenteral nutrition for a prolonged time due to short bowel and intestinal failure.

15. Treatment of AMI is optimal in a dedicated center using a focused care bundle and a multidisciplinary team. (Strong recommendation based on low-quality evidence 1C)

Recent published evidence suggests that treatment of occlusive AMI in “intestine stroke centers” using a multidisciplinary approach improves outcomes [142, 143]. Improving survival rates can be obtained if mesenteric ischemia is diagnosed and treated early. The goal of multidisciplinary approach is to keep the time to reperfusion as short as possible. The team often includes general surgeon (preferably an emergency surgery specialist), vascular surgeon, interventional radiologist, and intensivist. The concept of “intestinal stroke centers” has been promulgated in France and in China [144, 145].

Dedicated “intestinal stroke centers” have highlighted the effectiveness of a multidisciplinary approach focusing on: (1) removal of non-viable ischemic bowel, (2) preservation of intestine with revascularization, and (3) intensive care treatment to prevent progression to multiorgan failure. Utilizing this approach, Corcos et al. reported a 30-day survival of 95% in a small single-center study involving 18 patients presenting with occlusive AMI [146].

Recently, an implemented pathway and care bundle for patients with suspected AMI was introduced in Meilahti Helsinki University Hospital [18]. The key aspects of the “bundle” were elevated awareness, rapid diagnostics, and interventions including hybrid OR with endovascular treatment capacity. Patients treated under the bundle protocol were more often diagnosed with CT, had shorter mean in-hospital delay to operating room (median 3 h), and had revascularization done more often. The thirty-day mortality was lower in this group [17 (25%) compared with 23 (51%), *p* = 0.001] [18].

Well-designed multidisciplinary teams tend to optimize perioperative care for all involved patients. This includes patients with non-favorable prognosis. Efforts to improve surgical care should employ multidisciplinary teams to promote both quality and cost-effective care.

**Fig. 1:**
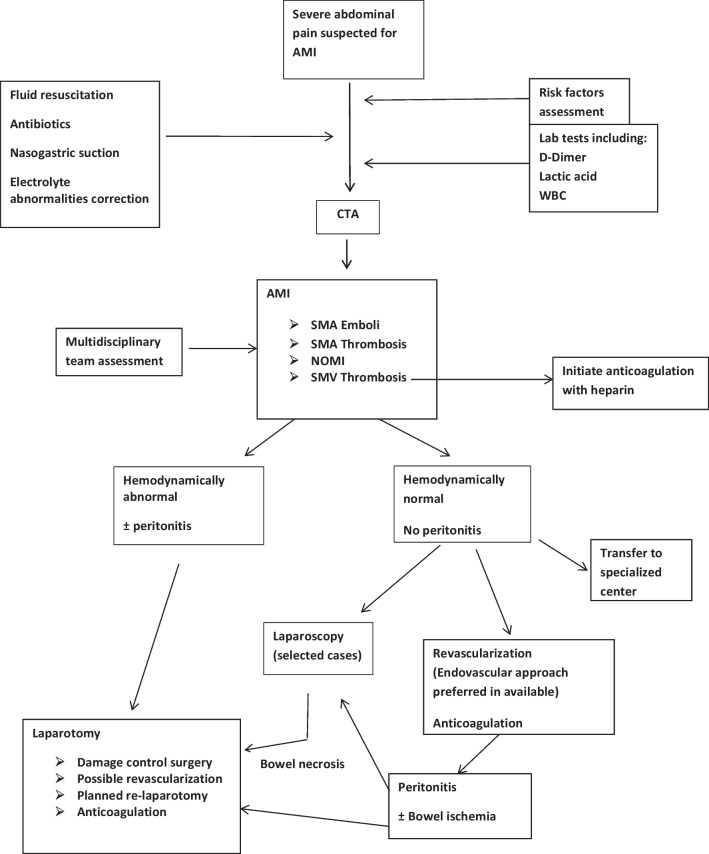
Management algorithm for patients with AMI

The management of patients with AMI is summarized in Fig. 1

16. Patients with short bowel syndrome following extensive bowel resection should have restoration of digestive continuity in association with hormonal therapy to optimize absorptive function and achieve nutritional autonomy. (Weak recommendation, low-quality evidence 1C)

The loss of large amounts of small bowel due to AMI can result in short bowel syndrome (SBS) and intestinal failure. SBS is associated with poor quality of life and a morbidity, which increases with age and comorbidities [147]. Management of patients with SBS can be challenging, especially in case of ostomies with associated large fluid losses and electrolyte imbalances [148]. Studies have shown that sparing the ileocecal valve and the colon is associated with nutritional independency in adults with SBS.

Recently, the use of synthetic growth agents such as the Glucagon-like peptide-2 (GLP-2) analog teduglutide has substantially changed the management of intestinal failure [149]. Multiple studies have shown that the use of a GLP-2 analog allowed a significant reduction of total parenteral nutrition (TPN) dependence and improved quality of life in patients with intestinal failure [150, 151].

Restoration of bowel continuity following extensive resection will improve functional outcome. If gastrointestinal tract reconstruction is not feasible, patients should be referred early for intestinal transplantation.

17. In case of massive gut necrosis, a careful assessment of the patients underlying comorbidities and advanced directives is advisable to find the optimal therapeutic strategy which could include palliation. (Weak recommendation, low-quality evidence 1C)

In cases of extensive infarction of most of the small bowel with or without a portion of the colon, the surgeon could face an ethical decision whether to do anything. Resection of the entire involved bowel will result in SBS with serious consequences.

The group from the Massachusetts General Hospital observed a decrease in the percentage of patients who underwent operative management of AMI over the last 25 years. This was correlated with an increase in the number of documented increased rates of “comfort measures only” status prior to surgical intervention from 50 to 70% [86].

Surgery may not be the best solution especially in elderly frail patients unable to tolerate long-term parenteral nutrition. In this regard, a preoperative discussion with the patient and their family is essential in guiding clinical decisions [152]. Shared decision making is very appropriate for this situation.

18. Patients undergoing revascularization should have surveillance imaging and long-term anticoagulation. (Strong recommendation based on moderate-quality evidence 1B)

The majority of patients treated for AMI will require lifelong anticoagulant/antiplatelet therapy to prevent relapse. In patients following endovascular stent placement, clopidogrel is administered for 6 months and acetylsalicylic acid as lifelong maintenance treatment. However, there is no scientific data on dual antiplatelet therapy after SMA stenting and the recommendation is based on experience from coronary interventions. When recovered following acute illness, most patients can switch to direct oral anticoagulants (DOACs) or vitamin K antagonists (VKA). Anticoagulation is given for 6 months, but most patients with underlying hypercoagulability should be considered for lifelong anticoagulation [113].

Continued surveillance for stent or graft restenosis is important, as AMI after mesenteric revascularization accounts for 6–8% of late deaths [153].

Patients undergoing revascularization should have surveillance imaging obtained via CTA or duplex ultrasound within 6 months, with frequent follow-up to enable early intervention for recurrent disease [135]. Current Society for Vascular Surgery guidelines recommend duplex ultrasonography at 1, 6, and 12 months after the intervention, and then annually thereafter [154].

Long-term care should be focused on the patient’s underlying medical comorbidities in order to minimize the risk of relapse. Lifestyle modification as well as management of hyperlipidemia, hypertension, and diabetes is necessary.

All established statements and recommendations are presented in Additional file 1: Table S3.

## Conclusions

AMI is a serious surgical emergency. The most important message is to have a high index of suspicion based on the combination of history of abrupt onset of abdominal pain, acidosis, and organ failure. This clinical scenario should prompt imaging (CTA) in order to establish the diagnosis. In parallel with rapid resuscitation and after careful assessment of the CTA, the patient should be explored to assess bowel viability, re-establish vascular flow, and resect non-viable bowel.

In the operating room, a focus on revascularization should take priority.

Classically open revascularization approaches have been used and described, in combination with damage control laparotomy. Recent developments, with improvement in early diagnosis, have allowed endovascular techniques to be implemented. Although evidence for the impact of endovascular interventions is limited at this time, they have apparent advantages over open surgery in some patients.

Preliminary evidence suggests that treatment of AMI in especially dedicated centers using a multidisciplinary approach improves outcomes. The evaluation and treatment of these patients by an interdisciplinary team reduce the time to reperfusion as short as possible.

## Supplementary Information

**Additional file 1: Table S3.** Summary of the updated 2022 guidelines for AMI: statements and recommendations.

## Data Availability

Not applicable.

## Abbreviations

AMI: Acute mesenteric ischemia
NOMI: Non-occlusive mesenteric ischemia
WSES: World Society of Emergency Surgery
CTA: Computed tomography angiography
SMA: Superior mesenteric artery
CMI: Chronic mesenteric ischemia
MVT: Mesenteric venous thrombosis
SMV: Superior mesenteric vein
VTE: Venous thromboembolism
tPA: Tissue plasminogen activator
I-FABP: Intestinal fatty acid-binding protein
alpha-GST: Serum alpha-glutathione S-transferase
CABA: Cobalt–albumin binding assay
MDCT: Multidetector computed tomography
MPR: Multiplanar reconstructions
DUS: Doppler ultrasonography
ICG: Indocyanine green
NIR: Near-infrared fluorophore
ICU: Intensive care unit
ROMS: Retrograde open mesenteric stenting
DCS: Damage control surgery
TEG, ROTEM: Viscoelastic techniques
PGE1: Prostaglandin E1
MOF: Multiorgan failure
aPTT: Activated partial thromboplastin time
LMWH: Low-molecular weight heparin
SBS: Short bowel syndrome
GLP-2: Glucagon-like peptide-2
TPN: Total parenteral nutrition
DOACs: Direct oral anticoagulants
VKA: Vitamin K antagonists
