# Supplementary material for: Acute mesenteric ischemia: updated guidelines of the World Society of Emergency Surgery
Source: World J Emerg Surg. 2022 Oct 19;17:54. doi: 10.1186/s13017-022-00443-x (PMC9580452; doi:10.1186/s13017-022-00443-x)
Supplement: Supplementary file 1 — Additional file 1: Table S3. Summary of the updated 2022 guidelines for AMI: statements and recommendations. [file 13017_2022_443_MOESM1_ESM.docx]

**Table 3. Summary of the updated 2022 guidelines for AMI: statements and recommendations.**

| **Primary assessment and diagnosis** |
| --- |
|  |
| 1. **Severe abdominal pain out of proportion to physical examination findings should be assumed to be AMI until disproven.** **(Strong recommendation based on low-quality evidence 1C)** |
| 1. **Clinical scenario and risk factors differentiate AMI as mesenteric arterial emboli, mesenteric arterial thrombosis, NOMI or mesenteric venous thrombosis.** **(Weak recommendation based on low-quality evidence 1C)** |
| 1. **Plain X-ray in not recommended in evaluating patients for intestinal ischemia (Strong recommendation based on moderate-quality evidence 1B)** |
| 1. **There are no laboratory parameters that are sufficiently accurate to conclusively identify the presence or absence of ischemic or necrotic bowel, although elevated l-lactate, leukocytosis and D-dimer may assist. (Weak recommendation based on moderate-quality evidence 2B)** |
| 1. **Computed tomography angiography (CTA) should be performed without delay in any patient with suspicion for AMI. (Strong recommendation based on high-quality evidence 1A)** |
| 1. **Nonocclusive mesenteric ischemia (NOMI) should be suspected in critically ill patients with abdominal pain or distension requiring vasopressor support and evidence of multiorgan dysfunction. (Weak recommendation based on low-quality evidence 2C)** |
| 1. **When the diagnosis of AMI is made, fluid resuscitation should commence immediately to enhance visceral perfusion. Electrolyte abnormalities should be corrected, and nasogastric decompression initiated. (Strong recommendation based on moderate-quality evidence 1B)** |
| 1. **Broad-spectrum antibiotics should be immediately administered. (Strong recommendation based on moderate-quality evidence 1C)** |
|  |
| 1. We recommend to maintain a high suspicion for AMI in patients presenting at ED admission with abdominal pain and signs of sepsis (Strength of recommendation High 1B) |
| 1. We recommend lactic acid monitoring in patients with the high suspicion of AMI (SoR High 1B) |
| 1. We recommend D-Dimer measurement to exclude intestinal ischemia (SoR High 1C) |
| 1. We recommend against the use of plain abdominal X-ray in evaluating patients for AMI (SoR High 1B) |
| 1. We recommend against delay of CTA in patients with clinical scenario and laboratory studies suspected for AMI (SoR High 1B) |
| 1. We recommend against delaying resuscitative manoeuvres, administration of empirical broad-spectrum antibiotic treatment and anticoagulant treatment with unfractionated heparin in patients with AMI (SoR High 1A) |
| 1. We recommend to investigate the intraabdominal pressure in critically ill patients ((SoR High 1A) |
| 1. We recommend for multidisciplinary approach in managing the patient with AMI (SoR High 1B) |
|  |
| **Operative and non-operative treatments** |
|  |
| 1. **Prompt laparoscopy/laparotomy should be done for patients with an overt peritonitis. (Strong recommendation based on low-quality evidence 1C)** |
| **Endovascular revascularization procedures are the primary option in cases of arterial occlusion when sufficient expertise is available. (Strong recommendation based on low-quality evidence 1C)** |
| 1. **Damage control surgery (DCS) with temporary abdominal closure is an important adjunct for patients who require intestinal resection allowing reassessment of bowel viability and in situations of severe abdominal sepsis. (Strong recommendation based on low-quality evidence 1B)** |
| 1. **Mesenteric venous thrombosis can often be successfully treated with a continuous infusion of unfractionated heparin.** **(Strong recommendation based on moderate-quality evidence 1B)** |
| 1. **When NOMI is suspected the focus is to correct the underlying cause and improve mesenteric perfusion. Infarcted bowel should be resected promptly. (Strong recommendation based on low-quality evidence 1C).** |
| 1. **Postoperative intensive care of AMI patients is directed towards the improved intestinal perfusion and the prevention of a multiple organ failure. (Strong recommendation based on low-quality evidence 1C)** |
|  |
| 1. We recommend against delaying laparotomy for hemodynamically unstable patients presenting with difuse peritonitis (SoR High 1A). |
| 1. We recommend laparoscopy as an alternative to laparotomy for intestinal viability assesment in selected cases in hemodynamically normal patients (SoR Weak 2C) |
| 1. We recommend for open or endovascular revascularization in patients with acute arterial mesenteric ischemia (SoR High 1B) |
| 1. We recommend for planned re- laparotomy in patients with intestinal ischemia after revascularization to reassess intestinal viability (SoR High 1B) |
| 1. We recommend intraoperative clinical judgment with Doppler ultrasonography in assessment of intestine viability (SoR Weak 1C) |
| 1. We suggest the use of Retrograde Open Mesenteric Stenting (ROMS) in AMI with arterial thrombosis when expertise, skills and resources are available (SoR Weak 2C) |
| 1. In tertiary centers, patients with AMI without signs of peritonitis, the step up approach (clinical - endovascular - laparotomy), should be encouraged as an alternative in order to restore the splancnic blood flow first. The lapatomy should be immidiately performed for damage control or stage therapy, in case of clinical deterioration (SoR High 1B). |
| 1. We can not recommend vasodilator therapy in patients with NOMI as an alterntive to surgical intervention ( SoR Weak 2C). |
| **Postoperative management** |
| 1. **Treatment of AMI is optimal in a dedicated center using a focused care bundle and a multidisciplinary team. (Strong recommendation based on low-quality evidence 1C)** |
| 1. **Patients with short bowel syndrome following extensive bowel resection should have restoration of digestive continuity in association with hormonal therapy to optimize absorptive function and achieve nutritional autonomy. (Weak recommendation, low- quality evidence 1C)** |
| 1. **In case of massive gut necrosis, a careful assessment of the patients underlying co-morbidities and advanced directives are advisable to find the optimal therapeutic strategy which could include palliation (Weak recommendation, low- quality evidence 1C)** |
| 1. **Patients undergoing revascularization should have surveillance imaging and long-term anticoagulation (Strong recommendation based on moderate-quality evidence 1B)** |
| 1. We recommend to manage patients presenting with intestinal ischemia in the ICU after surgery using principles of a multidisciplinary approach (SoR High 1B). |
| 1. We recommend for to administer intravenous unfractionated heparin as soon as possible for all patients with AMI. (SoR High 1B). |
| 1. We recommend for assessment of coagulation disorders and lifelong anticoagulation for all patients with MVT (SoR High 1B) |
| 1. We recommend for secondary cardiovascular prevention strategy in patients when recovered from AMI (SoR High 1B) |
| 1. We recommend for the transfer patients with AMI to the specialized centers that can offer a multidisciplinary assessment, both open and endovascular treatment and have hybrid operation facility (SoR High 1C) |
